# H_2_ Enhances Arabidopsis Salt Tolerance by Manipulating ZAT10/12-Mediated Antioxidant Defence and Controlling Sodium Exclusion

**DOI:** 10.1371/journal.pone.0049800

**Published:** 2012-11-21

**Authors:** Yanjie Xie, Yu Mao, Diwen Lai, Wei Zhang, Wenbiao Shen

**Affiliations:** 1 College of Life Sciences, Co. Laboratory of Nanjing Agricultural University, Nanjing, Jiangsu Province, China; 2 Carl Zeiss Far East, Nanjing Agricultural University, Nanjing, Jiangsu Province, China; Instituto de Biología Molecular y Celular de Plantas, Spain

## Abstract

**Background:**

The metabolism of hydrogen gas (H_2_) in bacteria and algae has been extensively studied for the interesting of developing H_2_-based fuel. Recently, H_2_ is recognized as a therapeutic antioxidant and activates several signalling pathways in clinical trials. However, underlying physiological roles and mechanisms of H_2_ in plants as well as its signalling cascade remain unknown.

**Methodology/Principal Findings:**

In this report, histochemical, molecular, immunological and genetic approaches were applied to characterize the participation of H_2_ in enhancing Arabidopsis salt tolerance. An increase of endogenous H_2_ release was observed 6 hr after exposure to 150 mM NaCl. Arabidopsis pretreated with 50% H_2_-saturated liquid medium, mimicking the induction of endogenous H_2_ release when subsequently exposed to NaCl, effectively decreased salinity-induced growth inhibition. Further results showed that H_2_ pretreatment modulated genes/proteins of zinc-finger transcription factor ZAT10/12 and related antioxidant defence enzymes, thus significantly counteracting the NaCl-induced reactive oxygen species (ROS) overproduction and lipid peroxidation. Additionally, H_2_ pretreatment maintained ion homeostasis by regulating the antiporters and H^+^ pump responsible for Na^+^ exclusion (in particular) and compartmentation. Genetic evidence suggested that *SOS1* and *cAPX1* might be the target genes of H_2_ signalling.

**Conclusions:**

Overall, our findings indicate that H_2_ acts as a novel and cytoprotective regulator in coupling ZAT10/12-mediated antioxidant defence and maintenance of ion homeostasis in the improvement of Arabidopsis salt tolerance.

## Introduction

Soil salinity is one of the most significant abiotic stresses and affects virtually every aspect of plant physiology and metabolism [1], [2]. Normally, salinity entails cellular hyperosmolarity and impairs ion homeostasis in plants. Secondary stresses such as oxidative damage thus occur as a consequence of the above primary effects [3]. The sustaining of a highly efficient antioxidant system, tightly regulated by different groups of transcription factors, becomes vital for plants to scavenge the salinity-triggered reactive oxygen species (ROS) overproduction [4], [5]. In Arabidopsis, the zinc-finger transcription factor ZAT10/12 plays a key role in abiotic stress tolerance, which could be attributed to the specific activation of antioxidant defence genes, e.g. *cytosolic ascorbate peroxidase1* (*cAPX1*) and *Fe superoxide dismutase* (*FSD1)* [3], [6], [7]. Recently, the role of heme oxygenase as a component of the antioxidant defence system has been well documented [8]. The mutation or over-expressing of Arabidopsis HO1 (HY1) exhibited salt hypersensitive or tolerance characteristics, respectively [9].

Several complementary lines of evidence have shown that enhanced Na exclusion and compartmentation, thereby maintaining a suitable ratio of K/Na, is crucial for plant salt stress adaptation [1], [10]. Two strategies, including Na^+^ efflux to the apoplast and vacuolar sequestration, appeared to be employed in Arabidopsis [2]. The Salt Overly Sensitive1 (SOS1) protein, which encodes a plasma membrane Na^+^/H^+^ antiporter and uses the driving force produced by plasma membrane H^+^-ATPase, plays an important role in Na^+^ exclusion. Mutation of *SOS1* led to a severely salt-sensitive phenotype due to impaired Na^+^ efflux [11], [12]. Additionally, Na^+^ can also be sequestered in vacuoles. Another type of Na^+^/H^+^ antiporter belongs to the Na^+^/H^+^ exchanger (NHX) family, which uses the proton gradient produced by vacuolar H^+^-translocating pyrophosphatase (AVP) to move Na^+^ into vacuoles. Constitutive overexpression of *NHX1* or *AVP1* increases Na^+^ accumulation in vacuoles and subsequently NaCl tolerance in Arabidopsis [13], [14].

Molecular hydrogen (H_2_) is the structurally simplest gas in nature and behaves as an inert gas at room temperature [15]. Recently, H_2_ was recognized as possessing antioxidative effects or modulating different signalling cascades by the rapid diffusion into tissues and cells, and exerting organ protective effects in clinical trials [16], [17], [18], [19], [20]. Normally, H_2_ production and assimilation are assumed to depend on the presence of a hydrogenase system in bacteria and green algae [21], [22]. Alongside these researches, earlier studies showed the evolution of H_2_ by seedlings, excised embryos, roots and hypocotyls in several higher plant species [23], [24], [25]. However, the enzyme(s) responsible for H_2_ production in higher plants remain uncertain. The experimental evidence and precise mechanism for H_2_ as a bio-effector or signalling molecule in plants upon multiple environmental stresses have hitherto been unknown.

The present study showed that salt stress induced an increase in endogenous H_2_ production in Arabidopsis seedlings. Furthermore, the preliminary mechanism of H_2_ when dissolved in liquid medium in improving plant resistance to salinity was investigated. The consumption of H_2_ has the potential to attenuate salinity toxicity by (1) modulating the expression of *ZAT10/12* and its regulated antioxidant genes/proteins, thus counteracting lipid peroxidation and ROS burst; and (2) maintaining the ion homeostasis by regulating the genes/proteins of antiporters and H^+^ pump responsible for Na^+^ exclusion (in particular) and compartmentation.

## Materials and Methods

### Plant Materials and Growth Conditions

*Arabidopsis thaliana capx1* [SALK_088596, Col-0] and *sos1* [CS3862, Col(gl1)] mutants were obtained from the Arabidopsis Biological Resource Center. Col-0 was used as the wild-type. Seeds were surface-sterilized and washed three times with sterile water for 20 min, then cultured in Petri dishes on solid Murashige and Skoog medium (MS, pH5.8) with 1% sucrose. Plates containing seeds were kept at 4°C for 2 d, and then transferred into a growth chamber with a 16/8 hr (23/18°C) day/night regime at 120 µmol m^−2 ^s^−1^ irradiation.

### Preparation of H_2_-saturated Aqueous Solution

Purified H_2_ gas was generated by using a H_2_-producing apparatus (Saikesaisi Hydrogen Energy Co Ltd, Shandong, China). In our experiment, H_2_-saturated aqueous solution was freshly obtained by pumping H_2_ gas through a polytetrafluoroethene filter to avoid bacterial contamination (300 ml/min). Then it was bubbled into 200 ml of sterilized water, or MS liquid medium (also regarded as H_2_-saturated aqueous solution) in the presence or absence of 150 mM NaCl for about 30 min, a sufficient duration to saturate the solution with H_2_. Then, the saturated 100% stock solution was immediately diluted to the required concentration (10, 25, 50, and 75% of saturation, [v/v]).

### Determination of H_2_ Production by Gas Chromatography (GC)

Arabidopsis endogenous H_2_ production was determined by GC (GC 5890C; Nanjing Kejie Technology Ltd, China) equipped with thermal conductivity detector (TCD) according to the method described by Ohsawa *et al.* [17] with modifications. The working conditions were optimized as TCD detector temperature at 100°C, 5Å molecular sieve as fixed phase, column temperature at 150°C, oven temperature at 60°C. Nitrogen gas was used as carrier gas and air pressure 0.2 MPa.

### Stress Treatments and Salt-tolerant Phenotype Analysis

5- or 25-day-old seedlings were selected for salt tolerance analysis. To test whether the NaCl-induced growth inhibition was alleviated by H_2_, pre-treatment, co-treatment, post-treatment or recovery treatment with the indicated saturations of H_2_ were applied in MS liquid medium. Seedlings without chemical treatments were the control (Con). The phenotypes, including fresh weight, chlorophyll content, and primary root growth, were observed at the indicated times [9], [26].

### Determination of the Content of Thiobarbituric Acid-reactive Substances (TBARS)

Lipid peroxidation of seedlings was estimated by measuring the amount of TBARS as previously described [27], [28].

### Histochemical Detection of H_2_O_2_ and O_2_^−^ Radical

H_2_O_2_ or O_2_^−^ level was measured by 3,3′-diaminobenzidine (DAB) or nitroblue tetrazolium (NBT) staining, respectively [29]. Seedlings were immersed in freshly prepared DAB solution (0.1% w/v, pH 3.8), vacuum infiltrated, and then incubated overnight in darkness at 22°C. Alternatively, seedlings were immersed in NBT solution in 10 mM potassium phosphate buffer (pH 7.8) containing 10 mM NaN_3_, vacuum infiltrated, and then incubated in darkness at 22°C for 1 h. After incubation, the stained seedlings were placed in a solution containing acetic acid:glycerol:ethanol (1∶1:3, v/v/v) at 95°C for 10 min, and then stored in 95% ethanol until photographed (model Stemi 2000-C; Carl Zeiss, Germany).

### Real-time RT-PCR Analysis

Total RNA was isolated using Trizol reagent (Invitrogen, Gaithersburg, MD, USA) according to the manufacturer’s instructions. Real-time quantitative RT-PCR reactions were performed using a Mastercycler® ep *realplex* real-time PCR system (Eppendorf, Hamburg, Germany) with SYBR® *Premix Ex Taq*™ (TaKaRa Bio Inc, China) according to the manufacturer’s instructions. Using specific primers (Table S1), relative expression levels of corresponding genes were presented as values relative to corresponding control samples at the indicated times or conditions, after normalization to *actin2/7* (accession number NM_121018) transcript levels.

### Determination of APX Activity

Arabidopsis seedlings (0.2 g) were homogenized in 5 ml of 50 mM potassium phosphate buffer (pH 7.0) containing 1 mM ethylenediaminetetraacetic acid (EDTA), 1% polyvinylpyrrolidone (PVP), and 1 mM ascorbic acid. The homogenate was centrifuged at 12 000 g for 15 min at 4°C, and the supernatant was immediately used for the APX activity determination by monitoring the decrease in *A*_290_ [27], [28]. Protein concentration was determined by the method of Bradford using bovine serum albumin as the standard [30].

### Western-blot Analysis for APX and H^+^-ATPase

Supernatant obtained for APX activity assays were also analyzed by western-blot. For western-blot analysis of H^+^-ATPase, Arabidopsis root tissues were collected and homogenized using 5 ml of ice-cold isolation medium containing 250 mM mannitol, 25 mM HEPES-Tris (pH 7.4), 1 mM EDTA, 1% PVP, 10% (v/v) glycerol, and 1 mM dithiothreitol. The whole procedure was performed at 4°C. The homogenate was filtered through four layers of cheesecloth and centrifuged at 1 300 g for 30 min. The supernatant was centrifuged at 60 000 g for 30 min to yield a crude membrane fraction for further suspension, which were used for western-blot analysis.

Twenty micrograms of protein were subjected to SDS-PAGE using a 12.5% acrylamide resolving gel (Mini Protean II System; Bio-Rad). Separated proteins were then transferred to PVDF membranes and nonspecific binding of antibodies was blocked with 5% BSA. The primary antibody against APX and plasma membrane H^+^-ATPase in Arabidopsis (Agrisera, Uppsala, Sweden) were applied overnight at 3000× dilution [31], [32], [33]. Immune complexes were detected using horseradish peroxidase (HRP)-conjugated goat anti-rabbit IgG. The color was developed with a solution containing DAB as the HRP substrate. Finally, the developed films were scanned (Uniscan B700^+^, Tsinghua Unigroup Ltd, Beijing China), and bands were analyzed by densitometry using Quantity One software (4.6.2 version).

### Determination of Ion Content

Na, K and Ca in seedlings were extracted and contents were measured by Inductively Coupled Plasma Optical Emission Spectrometry (Optima 2100DV; Perkin Elmer, USA) as described previously [34].

### Data Presentation and Statistical Analysis

Data are shown as means ± SE (standard error) from three independent experimental replications. Statistical analysis was performed using SPSS 16.0 software. Data are means ± SE from three independent experiments. Differences among treatments were analyzed by one-way ANOVA, taking *P*<0.05 level as significant according to *t*-test or Duncan’s multiple range test where appropriate.

## Results

### H_2_ Protected Arabidopsis from Salt Stress-induced Growth Inhibition

**Figure 1:**
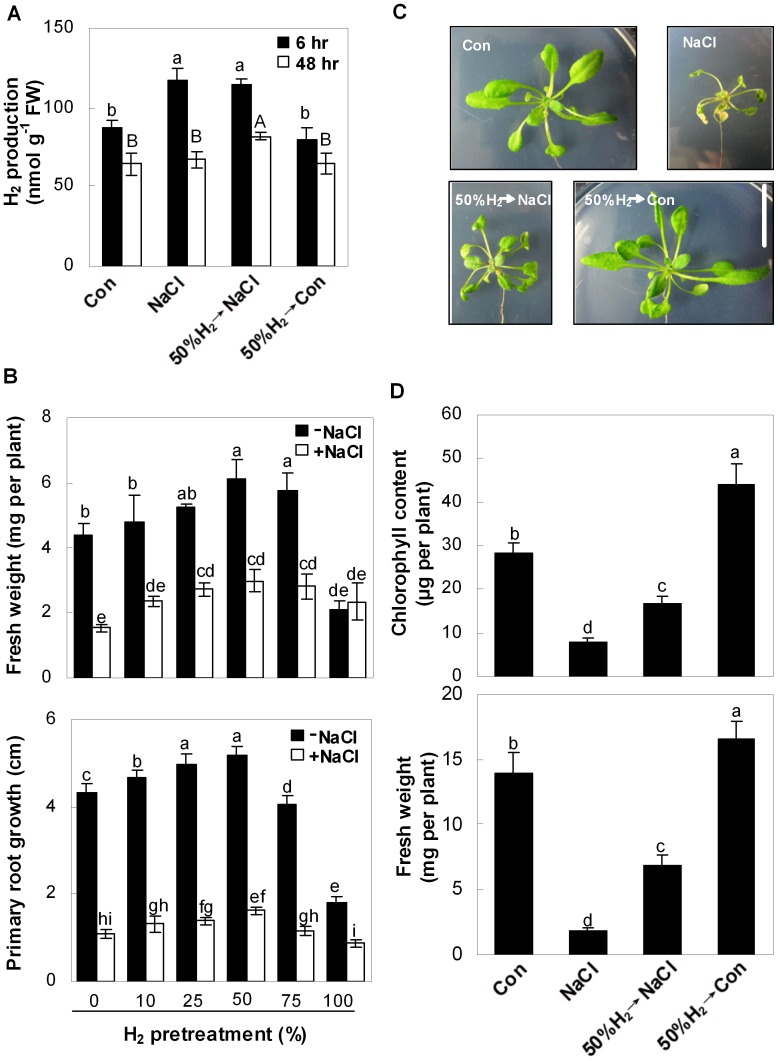
H_2_ alleviates salt stress-induced Arabidopsis seedlings growth inhibition. Effects of H_2_-saturated aqueous solution pretreatments with the indicated saturations for 24 hr on endogenous H_2_ production (A), fresh weight and primary root growth (B) in 5-day-old seedlings grown in the liquid MS medium with or without NaCl treatment for the indicated times (A) or 120 hr (B). Effects of H_2_ pretreatment on morphology (C), chlorophyll content and fresh weight (D) in Arabidopsis plants. 25-day-old seedlings were pretreated with or without 50% H_2_-saturated aqueous solution for 24 hr and then exposed to the liquid MS medium in the presence or absence of 150 mM NaCl for another 8 days. Sample without chemicals was the control (Con). Bar  = 2 cm (C). Statistical analysis was performed using SPSS 16.0 software. Data are means ± SE from three independent experiments. Bars with different letters are significantly different at *P*<0.05 according to Duncan’s multiple range test.

Previous studies demonstrated H_2_ production in seedlings and excised embryos [23], and our findings extended these observations. For example, in comparison with the NaCl-free control sample, H_2_ production in Arabidopsis seedlings increased significantly after 150 mM NaCl exposure for 6 h, and fell back to control level after 48 hr of treatment (Figure 1A).

To test whether endogenous H_2_ had any effect on the regulation of salt tolerance, a series of exogenous H_2_-saturated aqueous solutions with different levels of saturation were applied. Seedling growth was markedly inhibited after exposing 5-day-old Arabidopsis seedlings to NaCl for 5 d, as evaluated by changes in fresh weight and primary root growth (Figure 1B). Pretreatment with increased levels of H_2_-saturation in aqueous solution, in the range 10–50% saturation, led to considerable rescuing effects in a dose-dependent manner, with more significant performance in the development of leaf tissues compared to roots. A maximal inducible response was observed when 50% saturation of H_2_ was applied alone or followed by NaCl (in particular) (Figure 1B, S1); whereas higher concentrations (75 and 100%) were less effective. Therefore, 50% H_2_-saturated aqueous solution was used throughout this study. Similar ameliorating responses of H_2_ against the NaCl-induced growth inhibition were also observed in 25-day-old plants, as evaluated by morphology, chlorophyll content and fresh weight (Figure 1C, D, S2).

Interestingly, in comparison with controls, the pretreatment with 50% H_2_-saturated aqueous solution followed by NaCl stress for 6 and 48 hr significantly increased H_2_ production in seedlings, which also approximately mimicked a physiological response elicited by NaCl stress (Figure 1A). Whereas, slight but not significant decrease in H_2_ production was observed in plants pretreated with H_2_ aqueous solution alone. Our results clearly suggested that the cytoprotective effect of H_2_ was universal, which was supported by results obtained from the treatment of H_2_ and NaCl individually or simultaneously on seedling growth and chlorophyll content, showing that H_2_ pretreatment brought about the maximal responses (Figure S3). Taken together, these findings suggest beneficial roles of endogenous H_2_ in the attenuation of salinity toxicity.

### Alleviation of Lipid Peroxidation and ROS Homeostasis

**Figure 2:**
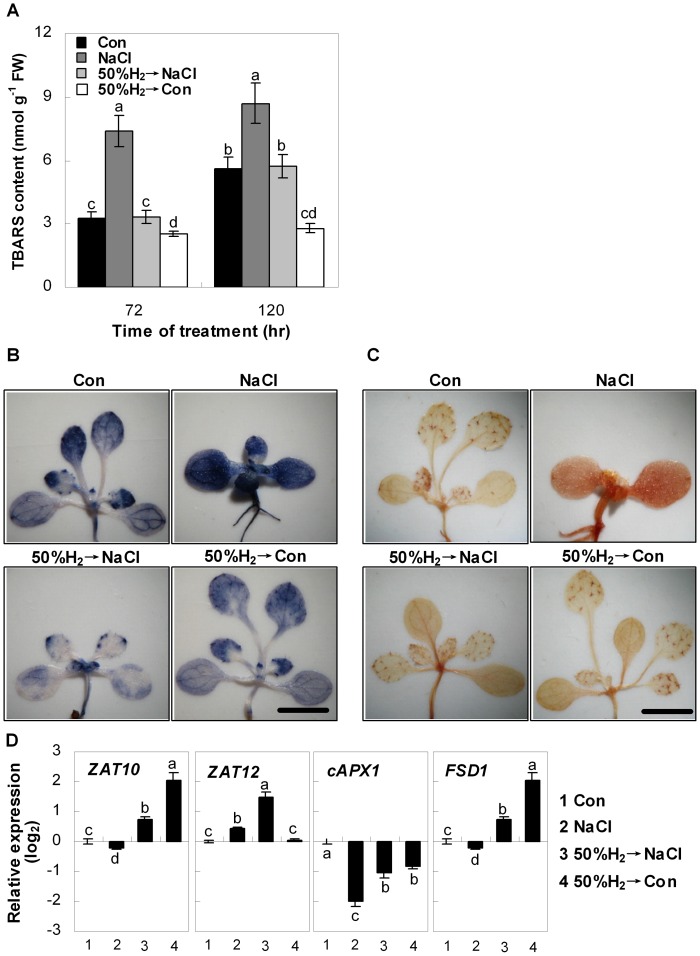
H_2_ protects Arabidopsis seedlings against salt stress-induced lipid peroxidation and ROS homeostasis. Seedlings were pre-incubated in 50% H_2_-saturated MS liquid medium for 24 hr, and then exposed to the MS liquid medium in the presence or absence of 150 mM NaCl. Sample without chemicals was the control (Con). Levels of lipid peroxidation (thiobarbituric acid reactive substance, TBARS) were measured at the indicated times (A). To detect O_2_^−^ and H_2_O_2_, seedlings were stained with NBT (B) and DAB (C) 120 hr after various treatments, respectively. Bar  = 2 mm. (D) Transcript levels of *zinc finger protein10* (*ZAT10*; At1g27730), *zinc finger protein12* (*ZAT12*; At5g59820), *cytosolic ascorbate peroxidase1* (*cAPX1*, At1g07890) and *Fe superoxide dismutase1* (*FSD1*, Ag4g25100) after 120 hr of indicated treatments were analyzed by real-time RT-PCR. Expression levels were presented as values relative to corresponding untreated control samples (Con), after normalization to *actin2/7* (At3g18780) levels. Statistical analysis was performed using SPSS 16.0 software. Data are means ± SE from three independent experiments. Bars with different letters are significantly different at *P*<0.05 according to Duncan’s multiple range test.

To assess whether the beneficial effects of H_2_ were related to oxidative stress, the TBARS formation, which is a reliable marker of lipid peroxidation and free radical generation, was measured. As expected, exposure of Arabidopsis seedlings to NaCl caused significant increases in the TBARS content over the 120 hr period (Figure 2A). However, pretreatment with 50%-saturated H_2_ aqueous solution produced a significant reduction in TBARS levels during similar time periods. Interestingly, H_2_ aqueous solution pretreatment alone also resulted in decreased TBARS levels, in comparison with the controls.

Salinity-induced ROS has been demonstrated to cause oxidative damage to plants, and O_2_^−^ and H_2_O_2_ are believed to be the most important components [5], [29]. The effect of H_2_ on the salinity-induced ROS overproduction was further investigated by histochemical staining. Basal levels with low production of O_2_^−^ (NBT staining) and H_2_O_2_ (DAB staining) were detected in control plants. However, the seedlings treated with NaCl were stained extensively in the hypocotyls (especially), cotyledons, and true leaves, whereas those pretreated with 50%-saturated H_2_ aqueous solution exhibited light staining (Figure 2B, C), all of which were consistent with the changes in TBARS content (Figure 2A). *In vitro* analysis further showed that H_2_ was able to directly quench H_2_O_2_ but not O_2_^−^ (Figure S4), which was in agreement with the results of DPPH (in particular) and TEAC assays but not FRAP assay (Figure S5).

### Modulation of Antioxidant Defence System

**Figure 3:**
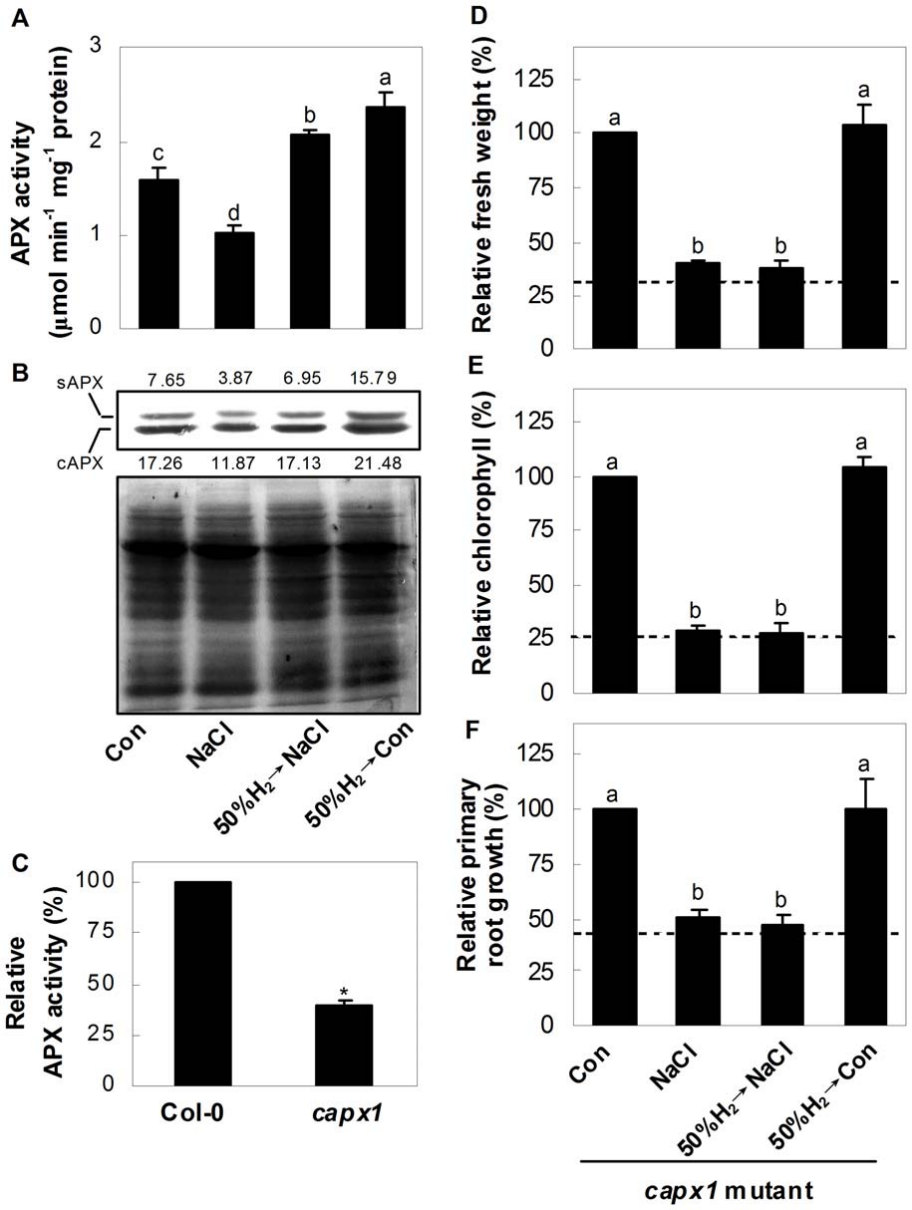
Modulation of APX by H_2_ and phenotypes of *cAPX1* knockout plants. (A) Changes of APX activity in Arabidopsis seedlings. (B) Up panel, stromal APX (sAPX) and cytoplasmic APX (cAPX) gene expression in Arabidopsis seedlings. Bottom panel, Coomassie Brilliant Blue-stained gels that showing equal amounts of proteins were loaded. The numbers above/below the band indicate the relative abundance of the corresponding sAPX/cAPX protein compared with that of the control sample. (C) Determination of total APX activity in 5-day-old wild-type and *capx1* mutant seedlings. (D–F) Changes of fresh weight, chlorophyll content, and primary root growth of *capx1* mutant seedlings. Corresponding samples without chemicals were regarded as a control (Con, 100%). 5-day-old wild-type and *capx1* mutant seedlings were pretreated with or without 50% H_2_-saturated aqueous solution for 24 hr, followed by the exposure to the liquid MS medium in the presence or absence of 150 mM NaCl for another 120 hr, and then phenotypic indicators were determined, respectively. The dashed lines denoted the inhibition rate of wild-type grown under NaCl stress, taking corresponding wild-type samples without chemicals as a 100%. Statistical analysis was performed using SPSS 16.0 software. Data are means ± SE from three independent experiments. Bars denoted by the different letters were different significantly at *P*<0.05 according to Duncan’s multiple range test (A, D-F). Additionally, the asterisk above the bar indicates significantly different in comparison with the wild-type at *P*<0.05 according to *t* test (C).

In order to get an insight into the *in vivo* function of H_2_, the expression of the ZAT10/12-related antioxidant defence system was analysed. Expression of corresponding genes was altered upon salinity stress (Figure 2D). Moreover, the pretreatment with 50%-saturated H_2_ aqueous solution weakened or blocked the down-regulation of *cAPX1*, *ZAT10* and *FSD1*, and strengthened the up-regulation of *ZAT12* gene expression, compared to salinity stress alone. The expressions of *ZAT10*, *cAPX1* and *FSD1* were altered by 50%-saturated H_2_ aqueous solution alone. To further determine whether protein synthesis was also involved in H_2_-modulated antioxidant defence system, the changes of total APX activity and its protein level in Arabidopsis seedlings were investigated. Results revealed that in comparison with the control samples, the salt-triggered inhibition of total APX activity and the reduced stromal APX (sAPX) and cytoplasmic APX (cAPX) protein level were clearly reversed by the pretreatment with 50%-saturated H_2_ aqueous solution (Figure 3A, B). However, in view of the inconsistency of APX activity and respect protein levels in Arabidopsis seedlings pretreated with 50%-saturated H_2_ aqueous solution followed by salinity stress (50%H_2_→NaCl), the possibility of post-translation regulation of APX triggered by H_2_ could not be easily ruled out.

Given the fact that cAPX1 is a central component of ROS network [35], the *capx1* mutant with reduced APX total activity (approximate 60%; Figure 3C) was adopted for further genetic analysis. In contrast to wild-type (Figure 1), it was found that no significant difference of salinity toxicity appeared in salinity stressed-*capx1* mutants in the presence or absence of H_2_ pretreatment (Figure 3D–F), further suggesting that cAPX1 might be the downstream target protein of H_2_ signalling.

We also noticed that in comparison with salt stress alone samples, there were no significant increases in the HY1 protein and expression of genes involved in the 2-Cys peroxiredoxins cycles (responsible for chloroplast-localized detoxification mechanisms) in H_2_-pretreated seedlings, suggesting that these were not involved in the H_2_-induced salt tolerance (Figure S6, S7).

### Reestablishment of Ion Homeostasis

**Figure 4:**
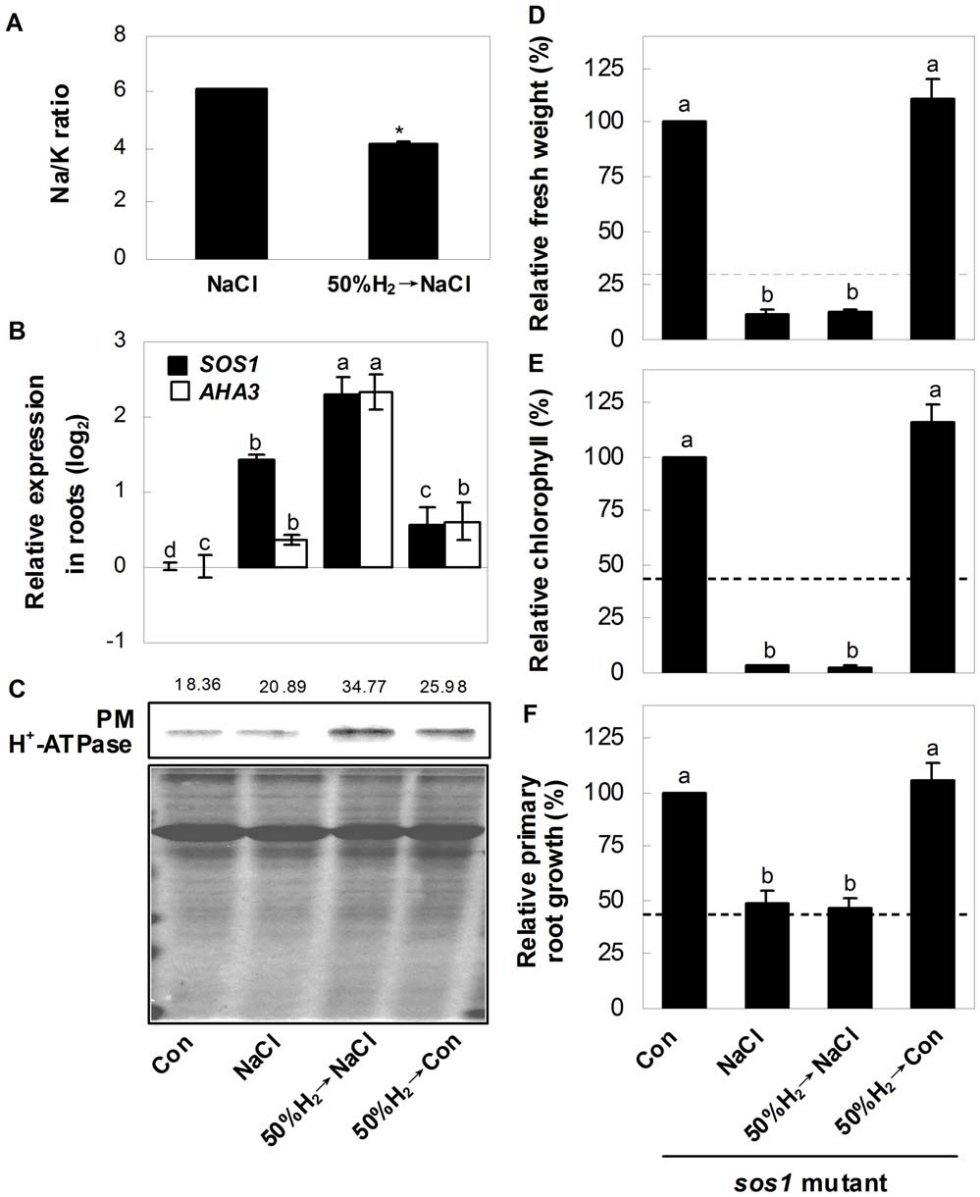
H_2_ regulates ion homeostasis and phenotypes of *sos1* knockout plants. (A) Changes of Na/K ratio in Arabidopsis seedlings. (B) Relative gene expression of *salt overly sensitive1* (*SOS1*; At2g01980), *Arabidopsis H^+^-ATPase3* (*AHA3*; At5g57350) in Arabidopsis seedling roots. (C) Up panel, plasma membrane (PM) H^+^-ATPase protein level in Arabidopsis seedling roots. Bottom panel, Coomassie Brilliant Blue-stained gels that showing equal amounts of proteins were loaded. The number above the band indicates the relative abundance of the corresponding H^+^-ATPase_protein compared with that of the control sample. (D-F) Changes of fresh weight, chlorophyll content, and primary root growth of *sos1* mutant seedlings. Corresponding samples without chemicals were regarded as a control (Con, 100%). 5-day-old wild-type and *sos1* mutant seedlings were pretreated with or without 50% H_2_-saturated aqueous solution for 24 hr, followed by the exposure to the liquid MS medium in the presence or absence of 150 mM NaCl for another 120 hr, and then phenotypic indicators were determined, respectively. The dashed lines denoted the inhibition rate of wild-type grown under NaCl, taking corresponding wild-type samples without chemicals as a 100%. Statistical analysis was performed using SPSS 16.0 software. Data are means ± SE from three independent experiments. The asterisk above the bar indicates significantly different in comparison with NaCl-treated alone sample at *P*<0.05 according to *t* test (A). Additionally, bars denoted by the different letters were different significantly at *P*<0.05 according to Duncan’s multiple range test (B, D–F).

The maintenance of ion homeostasis is crucial for plant survival upon salt exposure, so we compared elements ratios in H_2_-pretreated and non-pretreated salt-stressed plants. Stirringly, there was a notably decrease in Na/K ratio in H_2_-pretreated plants (Figure 4A), caused by the lower Na^+^ accumulation (Figure S8). However, no significant changes of K^+^ and Ca^2+^ contents were observed.

**Figure 5:**
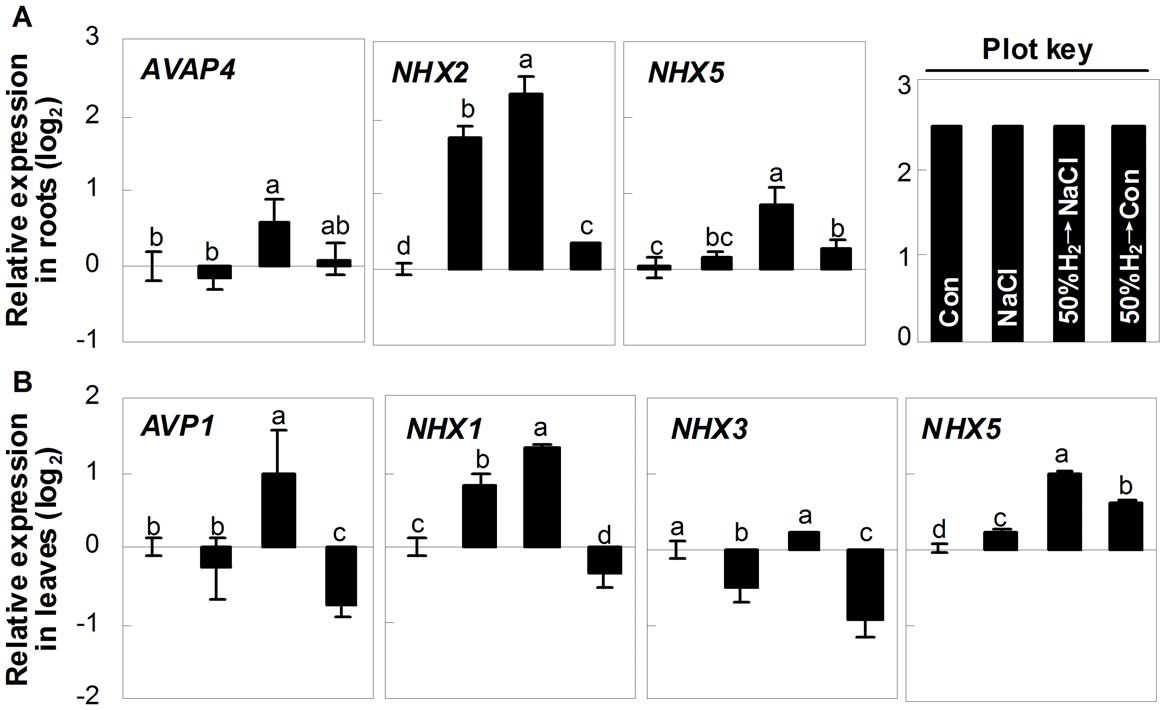
Regulation of transcripts responsible for Na compartmentation by H_2_. (A, B) Relative gene expression of *Arabidopsis V-type proton ATPase proteolipid subunit c4* (*AVAP4*; At1g75630), *sodium hydrogen exchanger2* (*NHX2*; At3g05030), *sodium hydrogen exchanger5* (*NHX5*; At1g54370), *Arabidopsis vacuolar membrane proton pump1* (*AVP1*; At1g15690), *sodium hydrogen exchanger1* (*NHX1*; At5g27150), and *sodium hydrogen exchanger3* (*NHX3*; At5g55470) in Arabidopsis seedling roots or leaves, respectively. Seedlings were pre-incubated in 50% H_2_-saturated MS liquid medium for 24 hr, and then exposed to the MS liquid medium in the presence or absence of 150 mM NaCl for anther 120 hr. Sample without chemicals was the control (Con). Plot key illustrated each bar shown in A and B. Statistical analysis was performed using SPSS 16.0 software. Data are means ± SE from three independent experiments. Differences among treatments were analyzed by one-way ANOVA, taking *P*<0.05 level as significant according to Duncan’s multiple range test.

We further examined the transcripts responsible for Na^+^ exclusion and compartmentation. The expressions of *SOS1* and *AHA3* in seedling roots, which were located in plasma membrane and controlled the Na^+^ exclusion, were up-regulated by salt stress. These tendencies were strengthened significantly by H_2_ pretreatment (Figure 4B). A similar result was also observed for the western blot analysis of PM H^+^-ATPase protein (Figure 4C). Further genetic evidence showed no significant difference in salinity toxicity in stressed-*sos1* mutant with or without H_2_ pretreatment (Figure 4D–F), further supporting the idea that *SOS1* might be the target gene of H_2_ signalling. Compared with salt stress alone samples, there were differential positive roles of H_2_ pretreatment in the gene expression of *AVAP4*, *NHX2* and *NHX5* in roots, *AVP1*, *NHX1*, *NHX3* and *NHX5* in leaves, further confirmed the potential regulatory role of H_2_ in Na^+^ sequestration (Figure 5). Additionally, the H_2_-pretreatment alone caused significant alteration of the above transcripts, except for *AVAP4*.

## Discussion

It has been proposed that biologically active gases possess advantages due to their low molecular weight, and can diffuse extremely rapidly into cells and likely to reach important target subcellular compartments. Thus, they have emerged as key regulator molecules and bio-effectors in multiple signalling events [36]. H_2_ is a colourless, tasteless, odourless, and nonirritating diatomic gas, and is generally regarded as physiologically inert in hyperbaric medicine. Recent studies in the clinical trials showed that H_2_ gas possessed protective effects in hepatic injury [37], myocardial infarction [38], and lipid and glucose metabolism in patients with type 2 diabetes [39]. In the present work, exposure of Arabidopsis seedlings to salt-stress caused rapid release of endogenous H_2_ (Figure 1A). In response to this observation, one question should be answered: what is the physiological significance of this endogenous H_2_ production? H_2_ production generally depends on the hydrogenase system in bacteria and green algae [21], [40], [41], [42]. Although previous results showed the existence of [FeFe]-hydrogenase-like protein in Arabidopsis [43], it was proposed that this hydrogenase-like protein in eukaryotes did not metabolize H_2_ [43], [44]. Thus, further work will be required to characterize the potential enzymatic or even non-enzymatic sources of endogenous H_2_ production in higher plants.

Regardless of these unsolved problems, the methodology using exogenous H_2_ provides a useful research tool to study the biological functions of H_2_, since its pretreatment mimicked a physiological response elicited by salt treatment (Figure 1A). Subsequently, our results illustrated that Arabidopsis plants pretreated with 50% H_2_-saturated aqueous solution exhibited significant relieved salt-induced growth inhibition (Figure 1B–D, S1, S2). Moreover, H_2_-induced recovery of seedling growth inhibition upon NaCl is unlikely to result from the direct reaction of NaCl with H_2_, as H_2_ pretreatment brought about the maximal rescuing responses (Figure S3). Intriguingly, this advantage of H_2_ was independent of plant growth stage, since this alleviation behaviour was observed in Arabidopsis plants at different growth stages (Figure 1, S1, S2).

In rat pheochromocytoma12 cultured cells, H_2_ specifically quenches detrimental ROS, such as •OH and peroxynitrite, while maintaining the metabolic oxidation-reduction reaction and other less-potent ROS, such as O_2_^−^ and H_2_O_2_ [17]. Interestingly, *in vitro* analysis showed a direct reaction of H_2_ with H_2_O_2_ but not O_2_^−^ (Figure S4). In the present study, H_2_ pre-treatment was performed to avoid the above interference. In view of the fact that this pre-treatment led to an increase in endogenous H_2_ production (50%H_2_→NaCl, Figure 1A), the possibility of H_2_ in direct quenching ROS could not be easily ruled out *in vivo*.

Multiple evidences illustrate that various abiotic stresses trigger ROS overproduction, thus leading to oxidative stress and cellular damage in plants [45]. It is also well established that transcription factor ZAT10/12 plays a key role in the specific activation of the ROS-related antioxidant system, including *cAPX1*, *cAPX2* and *FSD1* [7], which is partially correlated with plant salt tolerance [5]. Two lines of evidence supported the signalling role of H_2_: (1) H_2_ pretreatment could differentially prevent the salt-induced decrease of *ZAT10*, *cAPX1*, and *FSD1* transcripts, and strengthen the up-regulation of *ZAT12* expression (Figure 2D); (2) NaCl-induced inhibition of APX protein level and total activity was reversed by the pretreatment of 50%-saturated H_2_ aqueous solution (Figure 3A, B). These results indicated that the H_2_-regulated APX gene expression was, at least partially, contributed to mRNA and protein levels.

To further verify whether an “antioxidant molecular” or “signalling molecular” function plays the decisive effect in H_2_-enhanced Arabidopsis salt tolerance, the *capx1* mutant with disrupted antioxidant defence system was used [35]. Supposing that H_2_ operates as “signalling molecule” but not “antioxidant molecule”, feeding *capx1* mutant with H_2_ could rescue its salinity toxicity symptom. In our experimental conditions, however, genetic results showed that there was no significant difference in salinity toxicity in *capx1* mutant with or without H_2_ pretreatment (Figure 3D), further indicating that H_2_-induced protective role was mainly derived from its signalling molecular roles. Compared with the inhibition rates of wild-type (dashed lines), the *capx1* mutant appeared to be slightly salt tolerant (Figure 3D–F). Accordingly, Millet et al [46] reported that *apx1* mutant was tolerant to both salinity and osmotic stress. We supposed that these results might be attributed to the different compensated activation of other stress-responsive pathways by the mutation of *APX1* [47]. In our experimental conditions, considering that H_2_ pretreatment failed to rescue the salinity toxicity phenotype of *capx1* mutant, we hypothesized that the potential compensated pathway abovementioned is activated in a H_2_-independent fashion.

There was an expected correlation among transcripts of ZAT10/12-mediated antioxidant defence, H_2_O_2_ and O_2_^−^ content and lipid peroxidation (Figures 2, 3), and plant growth parameters (Figure 1, S1, S2). We further concluded that the cytoprotective role of H_2_ might be attributed to the activation of antioxidant defence signalling, and thereafter modulating ROS homeostasis and decreased TBARS formation. Clinical results showing that H_2_ is effective for many ROS-induced diseases are consistent with this proposition [48]. It was noteworthy that compared with the control samples, the expression of *ZAT10*, *cAPX1*, and *FSD1*, APX protein and activity were altered by 50%-saturated H_2_ aqueous solution alone (Figures 2D, 3A, B). Interestingly, treatment of 50%-saturated H_2_ aqueous solution alone also led to a series of significant changes, such as increased chlorophyll content and fresh weight (Figure 1), lowering TBARS content and ROS accumulation (Figure 2A–C). Although the identification of above mechanism goes beyond the scope of our work which discoveries H_2_-regulated Arabidopsis salt tolerance, it will be a part of future investigations.

Both the maintenance of ion homeostasis and the enhancement of antioxidant defence are two crucial mechanisms for plant survival upon salt stress [1], [5], [10], [28]. Regulation of Na^+^ exclusion and compartmentation, controlled by sodium exchanger located in plasma membrane and vacuole, are two effective strategies for plant to maintain ion homeostasis upon salinity stress [1], [10]. Meanwhile, the ability to retain K was also correlated with plant salt tolerance [49], [50]. Our results further showed that the biological benefit of H_2_ was attributed to its specifically reduced Na^+^ toxicity, whereas K^+^ and Ca^2+^ were not affected (Figure 4A, S8). Analysis of the genes and protein responsible for the function of Na^+^ exclusion in roots (Figure 4B, C) supported this proposition. These results also suggested that Na^+^ exclusion might play an important role in H_2_-enhanced Arabidopsis salt tolerance. Previous studies showed that the mutation of *SOS1* led to a severely salt-sensitive phenotype due to impaired Na^+^ efflux [11], [12]. Together with the slight salt-tolerant phenotype of *capx1* mutant (Figure 3), we further supposed that compared with the regulation of antioxidant defence system, the maintenance of SOS1-mediated Na^+^ exclusion seems more crucial, and thus appeared to be an effective strategy for plants survival. Our genetic evidence confirmed the stress hypersensitive phenotype of *sos1* mutant, and further showed no significant difference in salinity toxicity appearing in *sos1* mutant with or without H_2_ pretreatment (Figure 4D–F), suggesting that *SOS1* might be the downstream target of H_2_ signalling. Furthermore, our results illustrated that both reestablishment of ion homeostasis, especially Na exclusion, and the enhancement of antioxidant defence, are two indispensable and crucial strategies of H_2_-conferred plant salt tolerance. In view of the fact that the *AVP* and *NHX* transcript levels were also modulated by H_2_-pretreatment compared with salt stress alone (Figure 5), and the K^+^ content was not altered (Figure S8), the detailed mechanism of AVP- and/or NHX-mediated Na^+^ compartmentation, and SOS1-mediated Na^+^ exclusion (in particular) in H_2_-enhanced salt tolerance will be identified in the further study.

Together, in this work, we present evidence showing H_2_ participation in plant salt tolerance by modulation of ZAT10/12-mediated antioxidant defence, and reducing of Na^+^ toxicity by regulation of genes/proteins responsible for the function of Na^+^ exclusion (in particular) and possible compartmentation. This report opens a new window for the study of H_2_ in plant signalling, and provides a potential strategy for the improvement of plant salt tolerance. Further complementary genetic approaches are required to support the above conclusion, as well as to uncover the components of H_2_ signalling in plants.

## Supporting Information

Figure S1 **Morphology of salinity-stressed Arabidopsis seedlings pretreated by H_2_.** 5-day-old seedlings were pre-incubated in 50% H_2_-saturated MS liquid medium for 24 hr, and then exposed to the MS liquid medium in the presence or absence of 150 mM NaCl for anther 120 hr. Sample without chemicals was the control (Con). Bar = 1 cm.
(PDF)

Figure S2 **Morphology of Arabidopsis seedlings growth on media containing H_2_ and NaCl.** 25-day-old seedlings were pre-incubated in 50% H_2_-saturated MS liquid medium for 24 hr, and then exposed to the MS liquid medium in the presence or absence of 150 mM NaCl for anther 8 days. Sample without chemicals was the control (Con). Bar = 2 cm.
(PDF)

Figure S3 **Effects of pre-treatment, co-treatment, post-treatment or recovery treatment of H_2_ on NaCl-induced seedling growth inhibition and chlorophyll loss.** 5-day-old Arabidopsis seedlings were pre-incubated for 24 hr in the MS liquid medium saturated with or without H_2_ (50% saturation) or in the presence of 150 mM NaCl, followed by the incubation in the same medium containing 150 mM NaCl for another 120 hr, or 150 mM NaCl and 50% saturation H_2_ together for another 120 hr, or 150 mM NaCl for 48 hr followed by 50% saturation H_2_ plus NaCl for another 72 hr, or 150 mM NaCl for 48 hr followed by recovery in the MS liquid medium with or without H_2_ for another 72 hr. Afterwards, fresh weight (A) and chlorophyll content (B) were expressed relative to the corresponding data in the chemical-free control condition (only with MS; % control). Data are means ± SE from three independent experiments. Bars with different letters are significantly different at the *P*<0.05 level according to Duncan’s multiple range test.
(PDF)

Figure S4 ***In vitro*** **quenching abilities of 25 and 50% H_2_-saturated sterilized water to H_2_O_2_ (A) and O_2_^−^ (B).** Sterilized water was regarded as the control sample (Con). After 30 min of incubation, H_2_O_2_ content was determined by detecting the absorbance of the Fe^3+^-xylenol orange complex. Additionally, the specificity of H_2_O_2_ was tested by eliminating H_2_O_2_ in the reaction mixture containing catalase alone (CAT; 150U). O_2_^−^ was generated by the riboflavin system under illumination, and the photochemical reduction of NBT was monitored (Absorbance at 560 nm) after 5 min of incubation. Crude enzyme extract from leaves (leaf extraction from 25-day-old seedlings) were added as a positive control. Data are means ± SE from three independent experiments. Bars with different letters are significantly different at the *P*<0.05 level according to Duncan’s multiple range test.
(PDF)

Figure S5 **In vitro antioxidant activity of H_2_-saturated sterilized water and the well-known antioxidant ascorbic acid (AsA) determined by DPPH free radical-scavenging assay (A), TEAC assay (B), and FRAP assay (C).** Sterilized water was regarded as the control sample (Con). The concentrations of AsA were used at 0.5, 1, 5, 10, or 100 µg/ml, respectively. Data are means ± SE from three independent experiments. Bars with different letters are significantly different at the *P*<0.05 level according to Duncan’s multiple range test.
(PDF)

Figure S6 **Western blot analysis of HY1 protein.** Up panel, HY1 protein level in Arabidopsis seedlings. Bottom panel, Coomassie Brilliant Blue-stained gels that showing equal amounts of proteins were loaded. 5-day-old wild-type seedlings were pretreated with or without 50% H_2_-saturated aqueous solution for 24 hr, followed by the exposure to the liquid MS medium in the presence or absence of 150 mM NaCl for another 120 hr. Afterwards, samples were collected. The number above the band indicates the relative abundance of the corresponding HY1 protein compared with that of the control sample, calculated by Quantity One software (4.6.2 version).
(PDF)

Figure S7 **Effects of H_2_ pretreatment on the expression profile of** ***2-Cys peroxiredoxin A*** **(** ***2-Cys Prx A*** **, At3g11630),** ***2-Cys peroxiredoxin B*** **(** ***2-Cys Prx B*** **, At5g06290),** ***Thioredoxin x*** **(** ***Trx x*** **, At1g50320), and** ***NADPH-dependent thioredoxin reductase C*** **(** ***NTRC*** **, At2g41680) in Arabidopsis seedling leaves.** 5-day-old seedlings were pre-incubated in 50% H_2_-saturated MS liquid medium for 24 hr, and then exposed to the MS liquid medium in the presence or absence of 150 mM NaCl for anther 120 hr. Sample without chemicals was the control (Con). Data are means ± SE from three independent experiments. Bars with different letters are significantly different at the *P*<0.05 level according to Duncan’s multiple range test.
(PDF)

Figure S8 **K (A), Na (B), and Ca (C) contents in Arabidopsis seedlings.** Seedlings were pre-incubated in 50% H_2_-saturated MS liquid medium for 24 hr, and then exposed to the MS liquid medium in the presence or absence of 150 mM NaCl for anther 120 hr. Data are means ± SE from three independent experiments. Bars with different letters are significantly different at the *P*<0.05 level according to Duncan’s multiple range test.
(PDF)

Table S1 **The sequences of PCR primers for real-time RT-PCR.**
(PDF)

File S1 **Methods for Supporting Information.**
(DOC)
