# Supplementary material for: H2 Enhances Arabidopsis Salt Tolerance by Manipulating ZAT10/12-Mediated Antioxidant Defence and Controlling Sodium Exclusion
Source: PLoS One. 2012 Nov 21;7(11):e49800. doi: 10.1371/journal.pone.0049800 (PMC3504229; doi:10.1371/journal.pone.0049800)
Supplement: Figure S1 — Morphology of salinity-stressed Arabidopsis seedlings pretreated by H2. 5-day-old seedlings were pre-incubated in 50% H2-saturated MS liquid medium for 24 hr, and then exposed to the MS liquid medium in the presence or absence of 150 mM NaCl for anther 120 hr. Sample without chemicals was the control (Con). Bar = 1 cm. (PDF) [file pone.0049800.s001.pdf]

Figure S1.

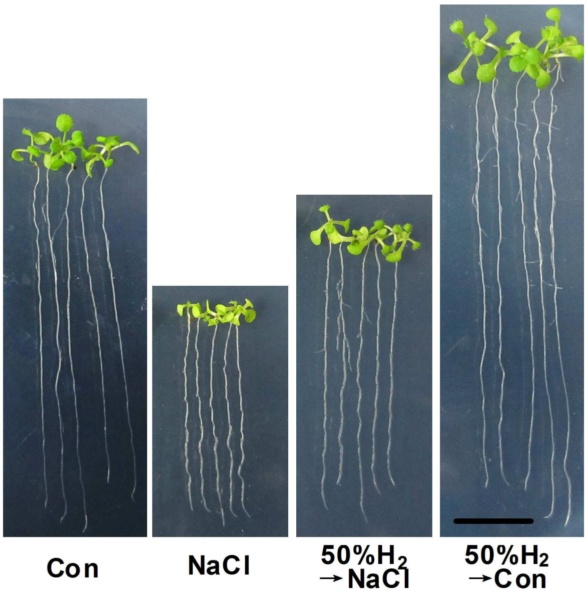

Figure S1. Morphology of salinity-stressed Arabidopsis seedlings pretreated by H_2. 5-day-old seedlings were pre-incubated in 50% H_2-saturated MS liquid medium for 24 hr, and then exposed to the MS liquid medium in the presence or absence of 150 mM NaCl for anther 120 hr. Sample without chemicals was the control (Con). Bar = 1 cm.
