# Supplementary material for: H2 Enhances Arabidopsis Salt Tolerance by Manipulating ZAT10/12-Mediated Antioxidant Defence and Controlling Sodium Exclusion
Source: PLoS One. 2012 Nov 21;7(11):e49800. doi: 10.1371/journal.pone.0049800 (PMC3504229; doi:10.1371/journal.pone.0049800)
Supplement: Figure S2 — Morphology of Arabidopsis seedlings growth on media containing H2 and NaCl. 25-day-old seedlings were pre-incubated in 50% H2-saturated MS liquid medium for 24 hr, and then exposed to the MS liquid medium in the presence or absence of 150 mM NaCl for anther 8 days. Sample without chemicals was the control (Con). Bar = 2 cm. (PDF) [file pone.0049800.s002.pdf]

Figure S2

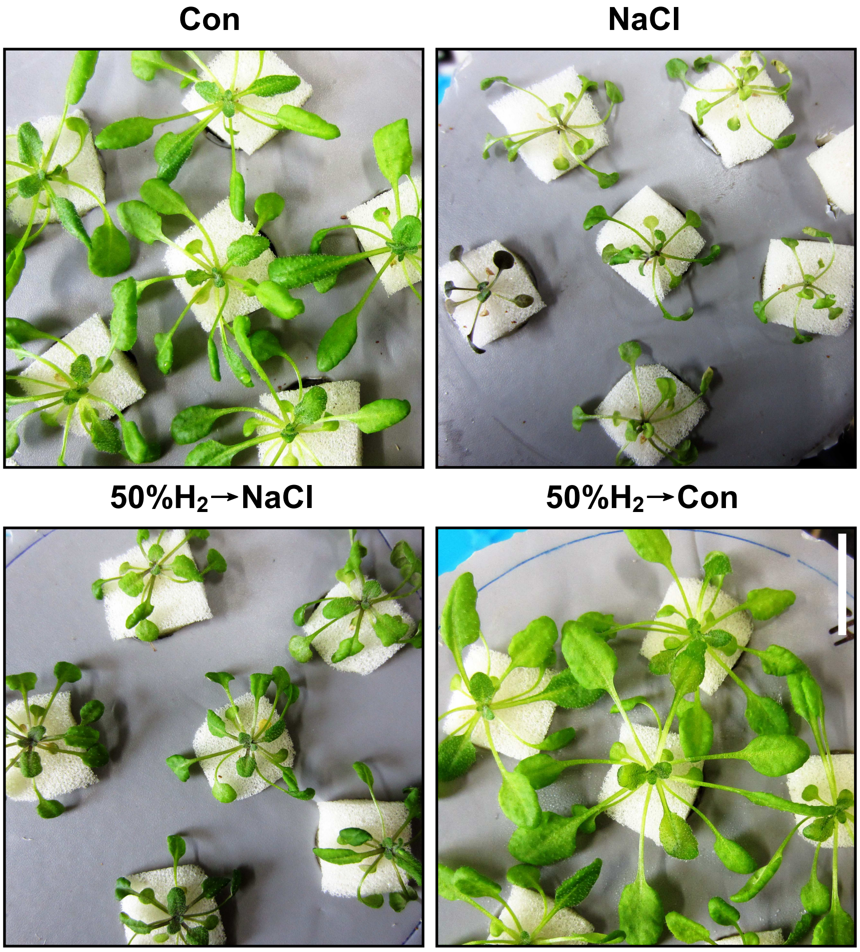

Figure S2. Morphology of Arabidopsis seedlings growth on media containing H_2 and NaCl. 25-day-old seedlings were pre-incubated in 50% H_2-saturated MS liquid medium for 24 hr, and then exposed to the MS liquid medium in the presence or absence of 150 mM NaCl for anther 8 days. Sample without chemicals was the control (Con). Bar = 2 cm.
