# Supplementary material for: H2 Enhances Arabidopsis Salt Tolerance by Manipulating ZAT10/12-Mediated Antioxidant Defence and Controlling Sodium Exclusion
Source: PLoS One. 2012 Nov 21;7(11):e49800. doi: 10.1371/journal.pone.0049800 (PMC3504229; doi:10.1371/journal.pone.0049800)
Supplement: Figure S3 — Effects of pre-treatment, co-treatment, post-treatment or recovery treatment of H2 on NaCl-induced seedling growth inhibition and chlorophyll loss. 5-day-old Arabidopsis seedlings were pre-incubated for 24 hr in the MS liquid medium saturated with or without H2 (50% saturation) or in the presence of 150 mM NaCl, followed by the incubation in the same medium containing 150 mM NaCl for another 120 hr, or 150 mM NaCl and 50% saturation H2 together for another 120 hr, or 150 mM NaCl for 48 hr followed by 50% saturation H2 plus NaCl for another 72 hr, or 150 mM NaCl for 48 hr followed by recovery in the MS liquid medium with or without H2 for another 72 hr. Afterwards, fresh weight (A) and chlorophyll content (B) were expressed relative to the corresponding data in the chemical-free control condition (only with MS; % control). Data are means ± SE from three independent experiments. Bars with different letters are significantly different at the P<0.05 level according to Duncan’s multiple range test. (PDF) [file pone.0049800.s003.pdf]

Figure S3

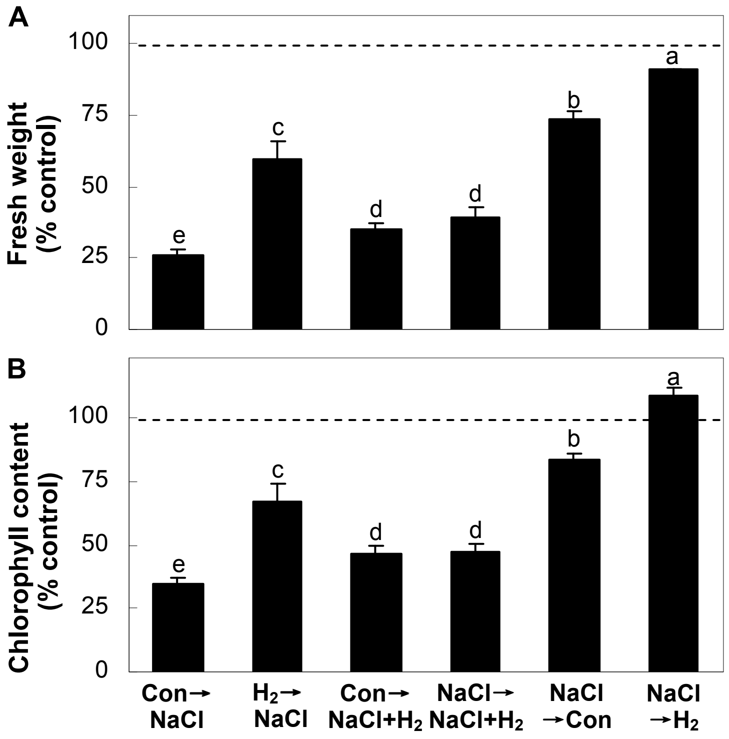

Figure S3. Effects of pre-treatment, co-treatment, post-treatment or recovery treatment of H_2 on NaCl-induced seedling growth inhibition and chlorophyll loss. 5-day-old Arabidopsis seedlings were pre-incubated for 24 hr in the MS liquid medium saturated with or without H_2 (50% saturation) or in the presence of 150 mM NaCl, followed by the incubation in the same medium containing 150 mM NaCl for another 120 hr, or 150 mM NaCl and 50% saturation H_2 together for another 120 hr, or 150 mM NaCl for 48 hr followed by 50% saturation H_2 plus NaCl for another 72 hr, or 150 mM NaCl for 48 hr followed by recovery in the MS liquid medium with or without H_2 for another 72 hr. Afterwards, fresh weight (A) and chlorophyll content (B) were expressed relative to the corresponding data in the chemical-free control condition (only with MS; % control). Data are means ± SE from three independent

experiments. Bars with different letters are significantly different at the $P < 0.05$ level according to Duncan's multiple range test.
