# Supplementary material for: H2 Enhances Arabidopsis Salt Tolerance by Manipulating ZAT10/12-Mediated Antioxidant Defence and Controlling Sodium Exclusion
Source: PLoS One. 2012 Nov 21;7(11):e49800. doi: 10.1371/journal.pone.0049800 (PMC3504229; doi:10.1371/journal.pone.0049800)
Supplement: Figure S4 — In vitro quenching abilities of 25 and 50% H2-saturated sterilized water to H2O2 (A) and O2− (B). Sterilized water was regarded as the control sample (Con). After 30 min of incubation, H2O2 content was determined by detecting the absorbance of the Fe3+-xylenol orange complex. Additionally, the specificity of H2O2 was tested by eliminating H2O2 in the reaction mixture containing catalase alone (CAT; 150U). O2 − was generated by the riboflavin system under illumination, and the photochemical reduction of NBT was monitored (Absorbance at 560 nm) after 5 min of incubation. Crude enzyme extract from leaves (leaf extraction from 25-day-old seedlings) were added as a positive control. Data are means ± SE from three independent experiments. Bars with different letters are significantly different at the P<0.05 level according to Duncan’s multiple range test. (PDF) [file pone.0049800.s004.pdf]

Figure S4

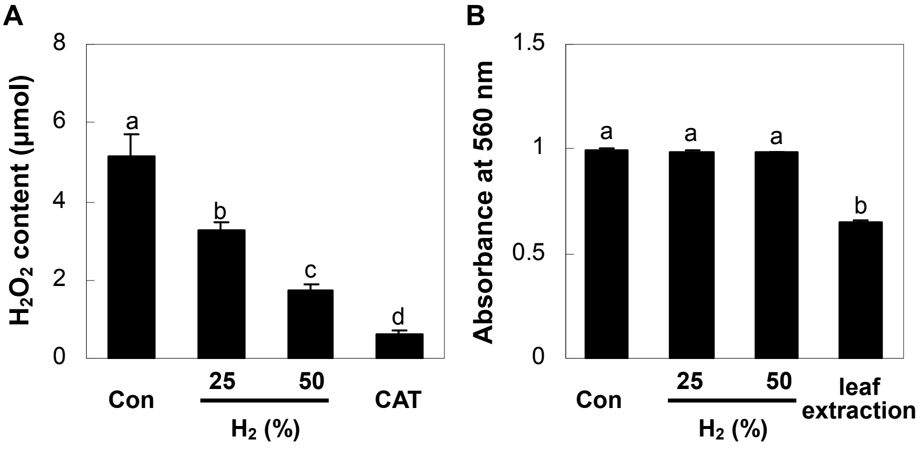

Figure S4. *In vitro* quenching abilities of 25 and 50% H_2-saturated sterilized water to H_2O_2 (A) and O_2^- (B). Sterilized water was regarded as the control sample (Con). After 30 min of incubation, H_2O_2 content was determined by detecting the absorbance of the Fe^{3+}-xylenol orange complex. Additionally, the specificity of H_2O_2 was tested by eliminating H_2O_2 in the reaction mixture containing catalase alone (CAT; 150U). O_2^- was generated by the riboflavin system under illumination, and the photochemical reduction of NBT was monitored (Absorbance at 560 nm) after 5 min of incubation. Crude enzyme extract from leaves (leaf extraction from 25-day-old seedlings) were added as a positive control. Data are means ± SE from three independent experiments. Bars with different letters are significantly different at the $P < 0.05$ level according to Duncan's multiple range test.
