# Supplementary material for: H2 Enhances Arabidopsis Salt Tolerance by Manipulating ZAT10/12-Mediated Antioxidant Defence and Controlling Sodium Exclusion
Source: PLoS One. 2012 Nov 21;7(11):e49800. doi: 10.1371/journal.pone.0049800 (PMC3504229; doi:10.1371/journal.pone.0049800)
Supplement: Figure S5 — In vitro antioxidant activity of H2-saturated sterilized water and the well-known antioxidant ascorbic acid (AsA) determined by DPPH free radical-scavenging assay (A), TEAC assay (B), and FRAP assay (C). Sterilized water was regarded as the control sample (Con). The concentrations of AsA were used at 0.5, 1, 5, 10, or 100 µg/ml, respectively. Data are means ± SE from three independent experiments. Bars with different letters are significantly different at the P<0.05 level according to Duncan’s multiple range test. (PDF) [file pone.0049800.s005.pdf]

Figure S5

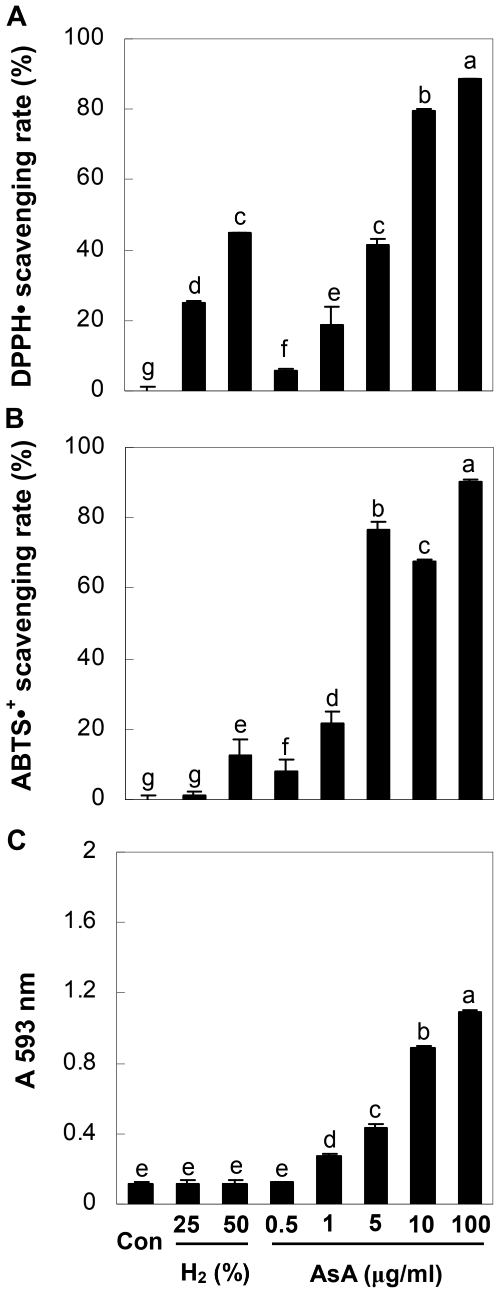

Figure S5. In vitro antioxidant activity of H_2-saturated sterilized water and the well-known antioxidant ascorbic acid (AsA) determined by DPPH free radical-scavenging assay (A), TEAC assay (B), and FRAP assay (C). Sterilized water was regarded as the control sample (Con). The concentrations of AsA were used at 0.5, 1, 5, 10, or 100 μg/ml, respectively. Data are means ± SE from three independent

experiments. Bars with different letters are significantly different at the $P < 0.05$ level according to Duncan's multiple range test.
