# Supplementary material for: H2 Enhances Arabidopsis Salt Tolerance by Manipulating ZAT10/12-Mediated Antioxidant Defence and Controlling Sodium Exclusion
Source: PLoS One. 2012 Nov 21;7(11):e49800. doi: 10.1371/journal.pone.0049800 (PMC3504229; doi:10.1371/journal.pone.0049800)
Supplement: Figure S6 — Western blot analysis of HY1 protein. Up panel, HY1 protein level in Arabidopsis seedlings. Bottom panel, Coomassie Brilliant Blue-stained gels that showing equal amounts of proteins were loaded. 5-day-old wild-type seedlings were pretreated with or without 50% H2-saturated aqueous solution for 24 hr, followed by the exposure to the liquid MS medium in the presence or absence of 150 mM NaCl for another 120 hr. Afterwards, samples were collected. The number above the band indicates the relative abundance of the corresponding HY1 protein compared with that of the control sample, calculated by Quantity One software (4.6.2 version). (PDF) [file pone.0049800.s006.pdf]

Figure S6

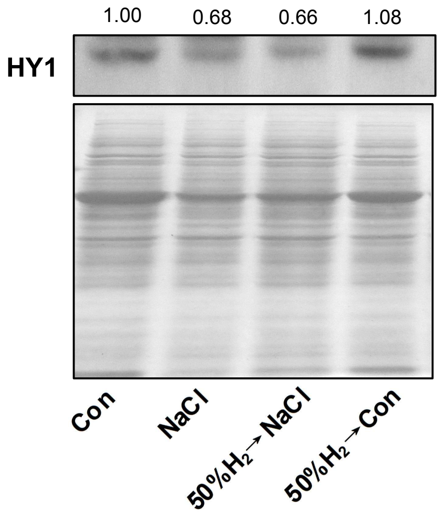

Figure S6. Western blot analysis of HY1 protein. Up panel, HY1 protein level in Arabidopsis seedlings. Bottom panel, Coomassie Brilliant Blue-stained gels that showing equal amounts of proteins were loaded. 5-day-old wild-type seedlings were pretreated with or without 50% H_2-saturated aqueous solution for 24 hr, followed by the exposure to the liquid MS medium in the presence or absence of 150 mM NaCl for another 120 hr. Afterwards, samples were collected. The number above the band indicates the relative abundance of the corresponding HY1 protein compared with that of the control sample, calculated by Quantity One software (4.6.2 version).
