# Supplementary material for: H2 Enhances Arabidopsis Salt Tolerance by Manipulating ZAT10/12-Mediated Antioxidant Defence and Controlling Sodium Exclusion
Source: PLoS One. 2012 Nov 21;7(11):e49800. doi: 10.1371/journal.pone.0049800 (PMC3504229; doi:10.1371/journal.pone.0049800)
Supplement: Figure S7 — Effects of H2 pretreatment on the expression profile of 2-Cys peroxiredoxin A ( 2-Cys Prx A , At3g11630), 2-Cys peroxiredoxin B ( 2-Cys Prx B , At5g06290), Thioredoxin x ( Trx x , At1g50320), and NADPH-dependent thioredoxin reductase C ( NTRC , At2g41680) in Arabidopsis seedling leaves. 5-day-old seedlings were pre-incubated in 50% H2-saturated MS liquid medium for 24 hr, and then exposed to the MS liquid medium in the presence or absence of 150 mM NaCl for anther 120 hr. Sample without chemicals was the control (Con). Data are means ± SE from three independent experiments. Bars with different letters are significantly different at the P<0.05 level according to Duncan’s multiple range test. (PDF) [file pone.0049800.s007.pdf]

Figure S7

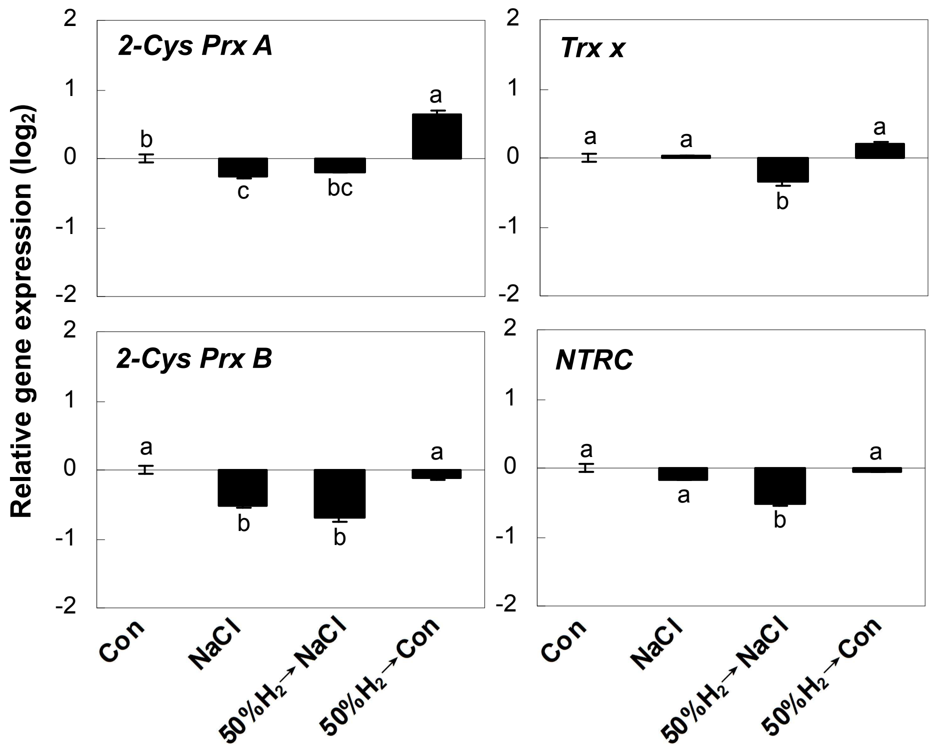

Figure S7. Effects of H_2 pretreatment on the expression profile of *2-Cys peroxiredoxin A* (*2-Cys Prx A*, At3g11630), *2-Cys peroxiredoxin B* (*2-Cys Prx B*, At5g06290), *Thioredoxin x* (*Trx x*, At1g50320), and *NADPH-dependent thioredoxin reductase C* (*NTRC*, At2g41680) in Arabidopsis seedling leaves. 5-day-old seedlings were pre-incubated in 50% H_2-saturated MS liquid medium for 24 hr, and then exposed to the MS liquid medium in the presence or absence of 150 mM NaCl for anther 120 hr. Sample without chemicals was the control (Con). Data are means ± SE from three independent experiments. Bars with different letters are significantly different at the $P < 0.05$ level according to Duncan's multiple range test.
