# Supplementary material for: H2 Enhances Arabidopsis Salt Tolerance by Manipulating ZAT10/12-Mediated Antioxidant Defence and Controlling Sodium Exclusion
Source: PLoS One. 2012 Nov 21;7(11):e49800. doi: 10.1371/journal.pone.0049800 (PMC3504229; doi:10.1371/journal.pone.0049800)
Supplement: Table S1 — The sequences of PCR primers for real-time RT-PCR. (PDF) [file pone.0049800.s009.pdf]

Supplementary Table 1. The sequences of PCR primers for real-time RT-PCR.

Primer name	Sequences (5'→3')
ZAT10-F	AGGCTCTTACATCACCAAGATTAG
ZAT10-R	TACACTTGTAGCTCAACTTCTCCA
ZAT12-F	TGTCCCATATGTGGAGTGGA
ZAT12-R	ATTGTCCACCATCCCTAGACT
cAPX1-F	ACTCTGGGACGATGCCACAAG
cAPX1-R	TCTCGACCAAAGGACGGAAAA
FSD1-F	GCTCGGCTCTTTCCCATTGC
FSD1-R	CAGCTTCCCAAGACACAAGATTGG
SOS1-F	CGTGAAGCAATCAAGCGGAAATT
SOS1-R	AAATTGGGTAGTGGATCCATTAACTATCAGA
AHA3-F	TCTGGCACAGAGAGAAGATGGCG
AHA3-R	TCTCCTCGAGTTTGTTGGGGCC
AVAP4-F	GCGATGATCAAACCATAAATACCTAACACA
AVAP4-R	CGGCGAAGAGTGGAGTTGGAGTT
NHX2-F	GCGCAATTCTGTGTGCCCTGAT
NHX2-R	CAGCATCAAGCAGCAAGTTGTGATC
NHX5-F	AGAAACAATGCCGGAGAGACCAAC
NHX5-R	GCAGGATTGGACACCGAGAATCTT
AVP1-F	GGGTCAAAACATCTACGGTGTGGAT
AVP1-R	GGAAACACCACTGCTGCTATTGGAA
NHX1-F	GTATGGATTTTGGGGTGTTGTCATCA
NHX1-R	CAGCACAGTGGTGTTTGGTATGCTG
NHX3-F	CACTTCCAGGCATGTATTTGCAATG
NHX3-R	CCCCAAAGCTTAAGCTGCTTGTCTT
2-Cys Prx A-F	ACTCTCATCTCTTCTCCC
2-Cys Prx A-R	AGGAGAAGAAAGGGTTCGAA

Primer name	Sequences (5’→3’)
2-Cys Prx B-F	CACCACCCTACTCTCTT
2-Cys Prx B-R	GGAAGAGGGTATGATTCTGG
Trx x-F	CTAGTTCGGTGATAAGATGC
Trx x-R	ATTGAGTTCAAGAGACCATC
NTRC-F	TCACCAACATGTGGCCC
NTRC-R	TTCTTCATCTTCACACCCGA
actin2/7-F	TCGTTTCGCTTTCCTTAG
actin2/7-R	CTTCACCATTCCAGTTCC
