# Supplementary material for: H2 Enhances Arabidopsis Salt Tolerance by Manipulating ZAT10/12-Mediated Antioxidant Defence and Controlling Sodium Exclusion
Source: PLoS One. 2012 Nov 21;7(11):e49800. doi: 10.1371/journal.pone.0049800 (PMC3504229; doi:10.1371/journal.pone.0049800)
Supplement: File S1 — Methods for Supporting Information. (DOC) [file pone.0049800.s010.doc]

**Methods for Supporting Information**

In vitro quenching ability determination

H_2_O_2_ content was determined by detecting the absorbance of the Fe^3+^-xylenol orange complex [1]. Also, O_2_^-^ was generated by the riboflavin system under illumination, and the photochemical reduction of NBT was monitored at absorbance of 560 nm [2].

In vitro antioxidant activity determination

Three methods, including DPPH (2,2-diphenyl-1-picrylhydrazyl) free radical-scavenging assay, TEAC (trolox equivalent antioxidant capacity) assay, and FRAP (ferric ion reducing antioxidant power) assay, based on reaction with electron –donating or hydrogen radicals producing compounds/antioxidants according to the reaction R+Aox-H→RH+Aox, were carried out according to the methods described [3,4].

Western-blot analysis for HY1

Arabidopsis whole seedlings were collected for western-blot analysis of HY1. The primary antibody against HY1 was made against the mature HY1 protein expressed in *E. coli*, with a molecular mass of 26.6 kDa [5]. Immune complexes were detected using horseradish peroxidase (HRP)-conjugated goat anti-rabbit IgG. The color was developed with a solution containing DAB as the HRP substrate. Finally, the developed films were scanned (Uniscan B700^+^, Tsinghua Unigroup Ltd, Beijing China), and bands were analyzed by densitometry using Quantity One software (4.6.2 version).

**References**

1. Wu M, Huang J, Xu S, Ling T, Xie Y, et al. (2011) Haem oxygenase delays programmed cell death in wheat aleurone layers by modulation of hydrogen peroxide metabolism. J Exp Bot 62: 235–248.

2. Han Y, Zhang J, Chen X, Gao Z, Xuan W, et al. (2008) Carbon monoxide alleviates cadmium-induced oxidative damage by modulating glutathione metabolism in the roots of *Medicago sativa*. New Phytol 177: 155–166.

3. Fu G, Zhang L, Cui W, Wang Y, Shen W, et al. (2011) Induction of heme oxygenase-1 with β-CD-hemin complex mitigates cadmium-induced oxidative damage in the roots of *Medicago sativa*. Plant Soil 345: 271–285.

4. Liu LX, Sun Y, Laura T, Liang XF, Ye H, et al. (2009) Determination of polyphenolic content and antioxidant activity of kudingcha made from *Ilex kudingcha* C.J. Tseng. Food Chem 112: 35–41.

5. Xie Y, Xu S, Han B, Wu M, Yuan X, et al. (2011) Evidence of Arabidopsis salt acclimation induced by up-regulation of *HY1* and the regulatory role of RbohD-derived reactive oxygen species synthesis. Plant J 66: 280–292.
